# Risk perception and sex behaviour in pregnancy and breastfeeding in high HIV prevalence settings: Programmatic implications for PrEP delivery

**DOI:** 10.1371/journal.pone.0197143

**Published:** 2018-05-14

**Authors:** Dvora Joseph Davey, Elise Farley, Catriona Towriss, Yolanda Gomba, Linda-Gail Bekker, Pamina Gorbach, Steven Shoptaw, Thomas Coates, Landon Myer

**Affiliations:** 1 Department of Biostatistics and Epidemiology, School of Public Health and Family Medicine, University of Cape Town, Cape Town, South Africa; 2 Department of Epidemiology, Fielding School of Public Health, University of California Los Angeles, Los Angeles, California, United States of America; 3 David Geffen School of Medicine, University of California Los Angeles, Los Angeles, California, United States of America; 4 The Desmond Tutu HIV Centre, University of Cape Town, Cape Town, South Africa; 5 David Geffen School of Medicine, Department of Family Medicine, University of California Los Angeles, Los Angeles, California, United States of America; The Ohio State University, UNITED STATES

## Abstract

HIV acquisition during pregnancy and breastfeeding significantly contributes toward paediatric HIV infection; however, little is known about risk behaviours in HIV-uninfected pregnant and postpartum women. We conducted twenty-six in-depth-interviews between July and December 2016 using a semi-structured interview guide among HIV-uninfected pregnant and recently postpartum women at-risk of HIV acquisition (defined as reporting ≥1 of the following: partner’s serostatus unknown or HIV-infected, recent condomless sex in pregnancy, and/or alcohol use during pregnancy) who attended primary healthcare services. Our study contextualizes factors related to risky sexual behaviours during pregnancy and postpartum periods and assesses knowledge and hypothetical acceptability of pre-exposure prophylaxis (PrEP) in pregnancy. Translated and transcribed data were coded and analysed by three researchers using a thematic analysis approach. In interviews with HIV-uninfected pregnant/postpartum women at-risk of HIV acquisition, we identified common themes associated with sexual risk behaviours during pregnancy, including: lack of control over decisions in sex and condom use in pregnancy, low perceived risk (e.g. beliefs that their partner has the same HIV-negative serostatus), and socio-cultural beliefs around condom use during pregnancy (e.g. contact with sperm is essential for baby’s development). PrEP knowledge was low among HIV-uninfected pregnant and breastfeeding women, and potential acceptability was good, though primary concerns were around the potential impact on the infant. While mothers presented a clear desire to protect themselves from HIV acquisition once pregnant, they also reported lack of control, and socio-cultural beliefs, like sex is good for the baby, that increased their risk of seroconversion. Mothers had limited PrEP awareness but reported hypothetical willingness to use PrEP because of concerns over HIV acquisition and onward mother to child transmission.

## Introduction

HIV-uninfected pregnant and breastfeeding women in South Africa are at high risk of HIV acquisition despite increased access to and initiation of antiretroviral therapy (ART) among those living with HIV [1–3]. A recent study in Kenyan discordant couples demonstrated that the HIV transmission probability was 1.05 per 1,000 sex acts when women were not pregnant, 2.19 in early pregnancy, 2.97 in late pregnancy, and 4.18 in postpartum women [4]. Hormonal changes that alter genital mucosal surfaces and/or the distribution of target cells at these surfaces could increase susceptibility during pregnancy and breastfeeding [3, 5, 6] Further, sexual behaviour increases risk of HIV acquisition during pregnancy and breastfeeding including frequent condomless vaginal and anal sex, multiple and concurrent sex partners, partners who have other sex partners during pregnancy/postpartum periods, substance use, and having an HIV-infected partner or a partner of unknown serostatus [7–11].

Increased susceptibility during pregnancy and breastfeeding could result from hormonal changes however, behavioural factors also play a role if male partners seeking other partners during pregnancy or postpartum bring HIV back into the relationship [3]. Maternal seroconversion during later pregnancy and breastfeeding contributes significantly toward paediatric HIV infections in South Africa [2]A mathematical model in South Africa demonstrated that HIV transmission from mothers seroconverting after their first antenatal visit accounts for approximately 34% of vertical HIV transmission[2]. In a recent meta-analysis, vertical transmission risk was significantly higher among women with incident vs. chronic HIV infection [3].

Considering the various risk factors that contribute to high HIV incidence in pregnancy and postpartum periods, effective use of pre-exposure prophylaxis (PrEP) could contribute to reducing maternal HIV acquisition, and hence vertical HIV transmission. PrEP could be used covertly by women during such periods. However, barriers currently exist to such PrEP use. Adherence to PrEP has been shown to be problematic among women in Southern Africa- particularly young women [12–14]. Further, there may be other barriers including perceived or real stigma associated with taking a drug for HIV when HIV-uninfected, especially among pregnant and breastfeeding women. While women’s concerns around PrEP use may be the same for pregnant women, there has been limited research on acceptability and adherence specifically among pregnant and breastfeeding women in sub-Saharan Africa where the burden of HIV is greatest.

Though pregnant women are not part of the target groups for PrEP delivery in South Africa, our study sought to assess the awareness and acceptability of PrEP as an option for pregnant women who are at substantial risk of HIV acquisition and vertical transmission during and after pregnancy. We conducted in-depth interviews among HIV-uninfected pregnant or recently postpartum women who were at risk of HIV acquisition because of self-reported risky sexual behaviours. Our qualitative study generated data to describe sexual behaviours and perspectives on hypothetical use of PrEP in this understudied group to better understand their knowledge and potential acceptability of PrEP in pregnancy.

## Methods

### Data collection

Between July and December 2016, we enrolled consenting pregnant or postpartum women attending primary care services in Cape Town South Africa into a study which included a survey on demographic and behavioural factors, including sexual behaviour during pregnancy and postpartum periods (n = 377). We analysed the results of the survey to identify a group of at-risk pregnant women who were HIV-uninfected and reported one or more risk factors for HIV acquisition, defined as reporting: (i) having a HIV-infected partner or partner with an unknown serostatus, (ii) any condomless sex during pregnancy, (iii) any alcohol use during pregnancy. Our risk score was adapted from previously validated risk assessment tools for identifying pregnant and postpartum women who may benefit from PrEP or predicting HIV acquisition over the course of one year in women [15–16]. Following this identification, the study interviewer called these participants to invite them to return to participate in an in-depth interview and counselled about the risks and benefits of participating in the study.

### Recruitment and eligibility

Study staff contacted 90 participants and requested them to return to the health facilities for an in-depth interview. Of the 90 contacted, 26 (29%) returned and signed an additional consent form for the interview. Most participants who returned were still pregnant (n = 19; 73%) and 7 women had delivered their infant by the time they were contacted to return for an in-depth interview We did not collect data on why women did not return. All women attended primary care services in Gugulethu Midwife Obstetric Unit.

Inclusion criteria was: (1) Confirmed HIV-uninfected (at study enrolment) (2) seeking antenatal or postnatal care in the health facility (3) ≥ 18 years old and (4) willing to participate in the study.

### Interview guides and procedures

Semi-structured interview guides were developed and translated into isiXhosa and back-translated into English for review. Two trained female interviewers conducted in-depth interviews about the following themes: (1) HIV risk perception, (2) risk behaviours (substance use and sex behaviour) during and after pregnancy, (3) discussion of HIV with partner and family/friends, (4) awareness of pre-exposure prophylaxis (PrEP), and (5)hypothetical acceptability of PrEP. and. Prior to asking about PrEP, interviewers explained that PrEP is “when HIV negative people use antiretroviral drugs, ARVs, to prevent them from getting HIV.” Participants were interviewed alone by interviewers matched in a private room within the health facility. Interviews were completed in the predominant local language, isiXhosa. The interviewers were previously trained, experienced interviewers who had conducted a previous qualitative study with our group. The interview lasted approximately 60-minutes. Participants received reimbursement for transportation (R20, approximately USD $1.50), a voucher (R80 from a supermarket, approximately USD $6.00) and a snack.

### Data analysis

Interviews were recorded, following which they were translated into English and transcribed by one of the study interviewers. The study coordinator reviewed all transcriptions and translations for accuracy and reviewed any inaccuracies with the interviewers, transcribers, or translators. We used inductive coding derived from the surveys and interviews. We used a thematic approach to our analysis, identifying key themes that were identified during the interviews derived from the data. [17] The interviewers and Study Coordinator were trained by the PI in qualitative interviews. The Study Coordinator took qualitative research courses and studied NVivo prior to the study. The interviewers did not have a relationship with participants before the interview. During the consent process, the participants were told that the goals of the study were to understand sexual behaviours, HIV knowledge and potential acceptability of new HIV prevention methods. reviewed and coded the transcripts into themes. DJD reviewed the findings and if there were discrepancies re-reviewed the transcripts together with the interviewer to come to an agreement. We used this technique to capture the meaning of their answers to their responses to the semi-structured interview questions. Coding was conducted using Nvivo software by the PI (DJD). We present the quotes by theme in and highlight the most relevant quotes from participants in the Results section. Supporting quotes are presented in table format by participant age and pregnant vs. postpartum status. We included some of the most relevant in-text quotations to give our participant voices a presence in the main text.

### Ethical approval

Our research was approved by the University of California, Los Angeles (IRB#16–001562) and University of Cape Town’s Institutional Review Board (IRB). Written Informed consent was obtained from the participants.

## Results

Participant characteristics are described in Table 1. Women’s median age was 24 (IQR = 21, 31) and 11 participants (42%) had completed secondary school. Most women reported that the father of the index pregnancy/child was a steady partner who did not live with them (n = 13, 50%), and most women were unemployed (n = 20, 77%). The monthly household income for 42% of participants was under R1000 (n = 11), and a further 42% was between R1001 and R5000 (n = 11). Almost all women reported having vaginal sex during pregnancy (96%) and about one-third reported >1 sex partner in the past 12-months.

**Table 1:** Demographic characteristics of participants (n = 26).

	*n / median*	*% / IQR*
Age	Median 24	IQR 21, 31
Education		
*Below secondary school*	14	54%
*Completed secondary school*	11	42%
*Degree/diploma*	1	4%
Relationship Status that best describes relationship with father of child		
*No relationship*	5	19%
*Married*	3	12%
*Steady partner living with me*	5	19%
*Steady partner not living with me*	13	50%
Employment Status		
*Employed fulltime*	6	23%
*Not Employed*	20	77%
Household Income Per Month		
*None*	1	4%
*<$80 per month*	11	42%
*$80-$400 per month*	11	42%
* >$400 ZAR per month*	3	12%
Previous Pregnancies	Median 1	IQR 0, 1
Live Children	Median 1	IQR 0, 1
Vaginal sex during pregnancy		
*Yes*	25	96%
Multiple partners in past year		
*Yes*	10	39%

The results of the in-depth interviews are presented in accordance to the four main themes related to the need for PrEP, and potential PrEP acceptability including: power differences, risk perception, socio-cultural beliefs, and PrEP knowledge and perceptions (Table 2).

**Table 2:** Quotes of HIV-uninfected pregnant women by theme.

**Power differences**	
I don’t know what his status (is) and I can’t just ask to go test. You have to give it time. Men like changing our minds as if they hypnotize us. You might have told yourself that you will use a condom and he changes your mind.	Pregnant, 22 yrs
If it was up to me, I would want us to use a condom when we have sex.	Pregnant, 34 yrs
I once suggested we use a condom, he got angry and asked why I am saying we should use a condom, he asked if I have a disease.	Postpartum, 21 yrs
I thought I was also infected. So when I tested and found out I don’t have it, that when we started using a condom. He did (have a problem using condoms) but I told him I will not infect my baby. I would rather break up with him.	Postpartum, 27 yrs
**Risk Perception**	
I tell him we need to use a condom because I can’t have sex without a condom if I keep on seeing numbers I don’t know on his phone. He will bring me AIDS.	Pregnant, 20 yrs
Yes, (my risk fluctuates) right now I am not even concerned (as I am not having sex as I have had my baby). I can’t get anything right now. I am not doing anything; how would I get it. It can’t put itself in me	Postpartum, 24 yrs
I would get it (HIV) because I don’t use a condom. I don’t know what else he does. I am not always with him.	Pregnant, 27 yrs
***Alcohol and other drug use***	
When you are drunk, yeah, when we are drunk and careless (you are highest risk of getting HIV). You just think about what you are going to do right now and don’t think of a condom.	Pregnant, 20 yrs
I would say the festive season and it is the riskiest time for HIV transmission, because people drink alcohol, and they take things lightly. They don’t see a reason for using a condom because they are drunk. And then you wake up in the morning, you don’t even know who you slept with and you didn’t use a condom. They want to be happy all time; happiness sometimes has bad results.	Pregnant, 27 yrs
(Riskiest time for HIV is) when you are drunk. yeah, when we are drunk and careless. Yeah. Drugs too. When your mind doesn’t focus on stuff. You just think about what you are going to do right now and don’t think of a condom.	Pregnant, 20 yrs
**Socio-cultural beliefs**	
***Condomless sex and baby’s health***	
We have that belief that when you are pregnant, you get horny. It might because that’s how the baby was conceived, and the baby just likes the sex. They say it hardens the baby’s fontanelle.	Pregnant, 20 yrs
I know that you need to (have sex) harden the fontanelle.	Pregnant, 22 yrs
He thinks if we have sex, the baby will look like him, the more we have sex, the more the baby will look like him.	Pregnant, 24 yrs
I believe that a male partner opens you up, preparing you for the labour	Pregnant, 24 yrs
Sperm helps the baby grow. When I told him we need to use a condom because I am pregnant. That I am at a high risk of getting diseases and the baby would also be at risk. He said that won’t happen because he needs to make the baby develop the way the baby was conceived.	Pregnant, 23 yrs
***Socio-cultural beliefs about sex in postpartum period***	
(You should wait) for 4 months (before having sex after birth) because the womb is not OK yet. They say that the baby will not be well, they will get diarrhoea, things like that	Pregnant, 31 yrs
Elder people say that you should wait 3 months because of the breast milk, that the child won’t grow…the elders know… but we must believe them because they know.	Pregnant, 24 yrs
People say that you must wait (to have sex) until the baby is old so that the baby is not disliked so that the baby doesn’t irritate people.	Pregnant, 21 yrs
If you have sex while breastfeeding, the baby loses weight.	Pregnant, 27 yrs
(You should wait) for 12 months (before having sex post birth). The reason is, I have seen babies that are not adorable and thin, when I ask my mother why that is. She said its having sex while you have a newborn. Woman like satisfying men, they should love their babies more than they love men (or else) they will have babies that are not adorable.	Pregnant, 23 yrs
They say you should not have sex before the baby is a year old, they say the baby will be slow	Postpartum, 19 yrs
**PrEP knowledge and potential acceptability**	
It’s a new drug and I don’t know what it will do. You know how sensitive babies are.	Pregnant, 20 yrs
It’s suitable because we know HIV is a lot here in South Africa. We should prepare ourselves. We don’t know what men will do the next day. I would say we will just have to take up this opportunity because it might change your whole life. I would advise that all the women in South Africa should use it (PrEP).	Pregnant, 20 yrs
I would not be sure how it would affect the baby… because its pills. I think it’s those with multiple partners (should take PrEP). Yes I would use it because they say a pregnant woman get infected easily. Even when breastfeeding, it would act as extra protection. They say breast milk has vitamins and nutrients for the baby so it would do the extra job on the side.	Pregnant, 32 yrs
I am not a pill person. Pills make me sick, especially you say it has side effects. But I would like to use it because I don’t think there is a person who does not want to protect themselves from HIV. I would be afraid of one thing. Maybe… I don’t know how ARVs look like… if I use it someone will think it’s ARVs	Pregnant, 22 yrs

### Power differences

Gender-based power differences within sexual relationships can negatively affect women's sexual and reproductive health. In our study, power differences affected risk behaviours such as condomless sex, not knowing the partner’s serostatus, and having multiple sex partners during pregnancy. HIV-uninfected pregnant and recently postpartum women in our study expressed high levels of risk perception and knew that they “should” use a condom to prevent HIV acquisition during pregnancy; however, most women reported that negotiating condom use was difficult, often felt like they did not have a say in the matter, and at times men showed firm resistance; “*I once suggested we use a condom*, *he got angry and asked why I am saying we should use a condom*, *he asked if I have a disease” (Postpartum*, *21 years old)*. This difficulty led to some respondents coming up with strategies to negotiate condom use such as withholding sex until a condom was used.

Women reported struggling to encourage their partners to get tested for HIV and often stated that their partners relied on the women’s HIV test to determine their own statuses, “*I don’t know what his status (is) and I can’t just ask to go test*. *You have to give it time* “(Pregnant, 22 years old). Women also reported blame being placed on them for the partner’s HIV status, “*Last year he tested and came back infected*. *He said I was the one who infected him*. *I had cheated*, *so I thought I should go test*. *I tested and found that I don’t have it*. *I told him that it’s obviously him because I don’t have it (HIV)” (Postpartum*, *27 years old)*. There was a sense of powerlessness around preventing HIV acquisition among pregnant women who suspected or knew that their partners had other partners. This, coupled with difficulty in negotiated condom use during sex in pregnancy, increases the risk of HIV acquisition for mother and baby. Lack of power to abstain from sex in the postpartum period was also expressed in interviews, with women reporting being fearful that their partner would leave them, or cheat on them, if they abstained from sex after birth. Women expressed the distrust that might arise if she abstains from postpartum sex, “*We are afraid that the men will leave us when having 3 months off of sex after birth*. *The contraceptive would not have entered my blood stream; he won’t trust why I suddenly want to use a condom*. *He won’t trust me*.*” (Pregnant*, *34 years)*.

### HIV risk perception

HIV risk perception was dynamic in nature and changed from pre-pregnancy to pregnancy and pregnancy to post-partum period. Changes from pregnancy to post-partum risk perception seem to hinge around the fact that after women give birth they often move into their mother’s house, during which a period of post birth abstinence ensues. As a result, women stated that they felt that they were at risk of HIV acquisition during pregnancy, but lower risk when abstinent. For example, one woman reported, *“Yes*, *my risk fluctuates right now I am not even concerned (as I am not having sex as I have had my baby)*. *I can’t get anything right now*. *I am not doing anything; how would I get it*. *It can’t put itself in me” (Postpartum*, *28 years old)*. Women shared that they perceived their risk of HIV acquisition to be high because of their partners’ infidelity or multiple partners; a lack of trust was also commonly shared. For instance, “I would get it (HIV) because I don’t use a condom. I don’t know what else he does. I am not always with him.” (Pregnant, 27 years) Others shared that their relationship status changed during or after pregnancy which changed their risk perception. For example, *“I felt I was at high risk of getting HIV when I first found out I was pregnant as the father of the baby had many girlfriends*. *I didn’t trust he was using a condom with them*. *He was not using a condom with me*, *so I got pregnant*. *Now I am at low risk as I have broken up with him*.*” (Pregnant 20 years)*

### Alcohol and drug use

Some women stated that drugs and alcohol increased their risk of HIV acquisition because their risk perception changed while on these substances, *“When you are drunk (you are at highest risk of getting HIV)*. *Yeah*, *when we are drunk and careless…*. *When your mind doesn’t (work)*. *You just think about what you are going to do right now and don’t think of a condom” (Pregnant*, *32 years old)*. The festive season (during Christmas/New Year’s) was stated as being a risky period because of consistent heavy alcohol and drug use, for instance,*” I would say the festive season is the riskiest time for getting AIDS*, *because people drink alcohol*, *and they take things lightly*. *They don’t see a reason for using a condom because they are drunk*. *And then you wake up in the morning*, *you don’t even know who you slept with and you didn’t use a condom*. *They want to be happy all time*, *happiness sometimes has bad results*.*” (Pregnant*, *27 years)*.

### Socio-cultural beliefs

Two main socio-cultural belief patterns emerged that may increase risk of HIV acquisition; the first was that male ejaculate is good for the baby’s growth and development, and pregnancy development, the second was socio-cultural beliefs around postpartum abstinence.

#### Condomless sex and baby’s health

Sperm was commonly reported as being good for the baby while in utero as it is believed that sperm assists with hardening the fontanelle and with the development of the foetus, *“We would just do it and we have that belief that when you are pregnant*, *you get horny*. *It might because that’s how the baby was conceived*, *and the baby just likes the sex*. *They say it hardens the baby’s fontanelle”*, *(Pregnant*, *20 years old) and “(I believe) that my baby will grow when I have sex*, *as the sperm is good for the baby” (Pregnant*, *32 years old*). Women reported that their partner prevented them from using condoms because of the belief that sperm was needed for the baby’s development. Many women reported that sex before birth was also good to prevent tearing during birth.

#### Post-partum abstinence

The second common belief was that it is important to abstain from sex after birth as sex may affect the baby’s health and beauty or attractiveness, “*You should wait 6-months after giving birth before you have sex*, *maybe even a year*. *Because the baby will not be well*. *The baby will get sick”* (Pregnant, 20 years old). Alternate reasons for post birth abstinence were that the baby will not be liked or accepted by the family, and that the mother should be looking after the baby and not focussing on romantic relationships, “*(You should wait) for 12 months (before having sex post birth)*. *The reason is… I have seen babies that are not adorable and thin if they have sex while having a new-born*. *Women like satisfying men*, *they should love their babies more than they love men (or else) they will have babies that are not adorable” (Pregnant*, *23 years old)*. Other women mentioned that they are expected to have frequent sex during pregnancy, but not after birth. For example, *“my beliefs are you should have sex with him (father of the baby) so that when you deliver the baby and you don’t experience any difficulty*. *But you shouldn’t have sex after birth because it makes the baby frail*. *I don’t want it*.*” (Post-partum*, *23 years old)*.

Post-partum abstinence may be associated with preventing another pregnancy close to the index pregnancy. Those beliefs may also help the mother focus on the health of her infant and breastfeeding, which would be limited if she were to get pregnant within the first-year post-birth. For example, one mother cited that reason for the health issues was reported as ‘dirt’ coming from the father’s ejaculate, “*I feel like I would be feeding the baby dirt (if we had sex before 6 months)*, *(the dirt would come) from the sex we would be having*, *it would (come from) the father’s sperm”* (Pregnant, 21 years old). The ‘dirt’ is believed to affect the baby through the breast milk.

### PrEP knowledge and potential acceptability

#### PrEP knowledge

Most mothers in our study did not know what PrEP was, *“No I have not heard of PrEP before” (Pregnant 20 years old)*. Another mother said, “*It’s a new drug*. *It is a drug and I don’t know what it will do*. *You know how sensitive babies are” (Pregnant*, *20 years old)*. One women knew a friend who had used PrEP, though her friend and her partner reported taking PrEP after sex, *“Yes I have heard of PrEP*, *my friend*, *she called it a ARV*. *She dated this guy who worked at the pharmacy*. *So*, *after sex*, *they would both take it*. *They would drink it only after sex*. *(HIV) doesn’t kill anymore but I would be protecting myself (by taking PrEP)*. *It would reduce the HIV… especially among the youth*.*”* (Pregnant, 22 years old) Another woman had participated in a PrEP study who reported that the Government should provide ART (or PrEP) to all to address the high HIV prevalence in South Africa: *“I was once in a study at Emavundleni*, *a HIV study…that study gave us ARVs to prevent HIV*. *They were still investigating*. *They eventually told us it was a success…*.*If the government would provide ARVs for everyone*, *HIV would decrease*, *and not infect people*.*” (*Post-partum, 27 years old)

#### PrEP potential acceptability

Despite limited knowledge about PrEP, all mothers interviewed, except for one mother, were interested in using PrEP as a method to prevent HIV acquisition during pregnancy and breastfeeding to prevent protect themselves and their baby from HIV acquisition. One mother stated “*(PrEP) sounds safe*. *Its suitable because we know HIV is a lot here in South Africa*. *We should prepare ourselves*. *We don’t know what men will do the next day*. *I would say we will just have to take up this opportunity because it might change your whole life*. *I would advise that all the women in South Africa should use it (PrEP)” (Pregnant*, *20 years old)*. Another mother said she would take PrEP during pregnancy or breastfeeding to prevent HIV “*as long as I know what it’s for and how it will help me” (Pregnant*, *25 years old)*. One postpartum mother in a serodiscordant relationship acknowledged the need for PrEP as her partner was not taking ARVs, *“I would be happy (to take PrEP) because the father of my baby doesn’t take his HIV treatment*. *We only rely on the condom*, *so I am at high risk*. *nothing would concern me (about taking PrEP)*. *I am concerned now*, *relying only on a condom*. *I am always worried if the condom will break*. *It used to break before*, *if it breaks now*, *I will be more concerned*.*” (Post-partum 27 years)*

However, many mothers raised concerns about the fact that PrEP may not be effective, or may be harmful, *“because this is the first time I hear about it*, *It (may not) really work*. *I will be worried that I will think I will stay negative and I might get infected” (Pregnant*, *25 years old)*.

The primary concern about taking PrEP during pregnancy and breastfeeding was the potential impact on the infant. Another mother stated, *“because it’s a pill… I would not be sure how it would affect the baby”* (*Pregnant*, *22 years old)*, and other mothers reported they would only take PrEP if it doesn’t have an effect (or side effects) on the baby. Other questions were raised around safety for themselves, “*I would be worried about how it will treat me and would make me sick” (Pregnant*, *27 years old) or “I am avoiding taking pills (while pregnant)” (Pregnant*, *25 years old)*. An important issue of stigma was raised by one women who said she would not take PrEP. She said: “*I wouldn’t feel free…*. *I would be ashamed*. *I would be afraid that people would avoid contact with me because they think I am sick because I am drinking pills*. *Maybe the father of my baby would avoid contact with me…*. *friends and the father of my baby would not understand that*. *They would think I am hiding something*.*”* Some mothers stated that they would be more willing to use PrEP during breastfeeding; for example, “*No*, *the baby would out of the tummy*. *I would use it (PrEP)…*.*and the breast milk is healthy*, so it will destroy all that sh*t. *It would be good*.*”* (Post-partum, 24 years old)

## Discussion

This study demonstrated that HIV-uninfected pregnant women and new mothers presented a clear desire to protect themselves from HIV acquisition once pregnant; however, they also reported lack of control, and socio-cultural beliefs, for example that frequent condomless sex is good for the baby, that increased their risk of HIV acquisition. Mothers had limited PrEP awareness but reported willingness to use PrEP because of concerns over HIV exposure during pregnancy and postpartum periods. Mothers expressed changing risk perception and sex behaviours during pregnancy and postpartum periods which may affect when a woman would need to take PrEP to prevent HIV acquisition. For example, since it appears that the frequency of sexual activity declines in the post-partum period as opposed to during the pregnancy, PrEP could be prioritized for use during the pregnancy as compared to the early post-partum period when most women were abstinent. Gender based power differences within sexual relationships were mentioned by most women as the most common factor that led to inability to use condoms consistently. This gender imbalance may be greater during pregnancy when the woman is more vulnerable to HIV acquisition and dependent on the father of the child emotionally and financially [18–19]Very few women in the study reported using condoms whilst pregnant, even when they perceived there may be a risk they would get infected with HIV. Socio-cultural beliefs could contribute to increasing HIV risk, such as the belief that condomless sex made the baby healthy, though other beliefs could be protective, for example the belief that sex during breastfeeding is “dirty” which may contribute toward increasing postpartum abstinence. Improved understanding of drivers of risky sex during pregnancy is essential to delivery of HIV prevention interventions in pregnancy and postpartum periods, including PrEP delivery.

Of concern were the overwhelming reporting of gender-based power differentials in this and other studies in the region [18, 20, 21]that limit women’s ability to protect themselves from exposure to HIV and other sexually transmitted infections. In South Africa, gender-based violence and rape are prevalent, including in pregnant women [18]. This study confirms the need for interventions that address power differentials, including couples counselling and testing for HIV, and interventions that prevent gender-based violence. In a context of limited power over decisions over sex and condom use, female-controlled interventions such as female condoms and PrEP are essential to prevent HIV acquisition during pregnancy. A recent qualitative study in Kenyan serodiscordant couples found that couples held the HIV-infected individual as responsible for HIV prevention and women accountable for prevention methods such as condom use [22].Understanding what motivates pregnant and breastfeeding women to use PrEP to prevent HIV is critical. Like Patel et al’s study, we agree that HIV prevention interventions must address important gender norms that may pose barriers to PrEP uptake. Further, including male partners in couple’s HIV counselling and testing, and PrEP counselling may help address gender norms that may hold women undeservedly responsible for prevention of HIV transmission.

Participants in our study reported alcohol use as decreasing inhibition and self-control. This finding is supported by prior research which demonstrated that alcohol and drugs were associated with risky sexual behaviour among pregnant women [23]Heavy alcohol use in women has also been found to increase risk of HIV acquisition, and delay ART initiation among HIV-infected African women [24]. Heavy alcohol use in pregnant and breastfeeding women is common in South Africa and will have an impact on PrEP delivery and adherence. HIV prevention interventions, including PrEP promotion and delivery, need to address alcohol use as a key barrier to uptake and adherence. For example, counsellors and nurses in ANC should be trained to ask questions about alcohol and other drug use during counselling sessions to assess the risk of HIV acquisition, transmission or fetal alcohol syndrome.

Our findings regarding the impact of socio-cultural beliefs around condomless sex, and pressure from other people around sexual behaviours during pregnancy and breastfeeding periods are supported by other African studies. Rogers et al. found that couples shared health related beliefs including that sperm was good for the baby’s growth and hardening of the fontanelle [21]. Previous studies have similarly demonstrated the belief that women should wait at least three months after birth before having sex to ensure that the baby was healthy and seen as ‘adorable’ [21] The concept of body image, including infant body image, has been the subject of numerous sociological approaches [25] Anthropological studies have reviewed the role of parents, including fathers and in-laws, in affecting the body image, or attractiveness, of their infant. This impacts on breastfeeding and nutrition of the infant [26]. Programs and PMTCT guidelines should acknowledge the role of concepts around body image and other socio-cultural beliefs to build off them to encourage condom use when postpartum sex resumes and educate women about the increased risk of HIV acquisition in condomless pregnancy and postpartum sex [3].

Our study has critical implications for the design and delivery of HIV prevention interventions, including PrEP (e.g. vaginal rings, injectables, etc.), to HIV-uninfected pregnant and breastfeeding women at risk of HIV acquisition. Considering the fluctuating sexual risk during pregnancy and postpartum periods, lack of control, and socio-cultural beliefs around condomless sex that limit the use of condoms during sex, additional female controlled HIV prevention methods are urgently needed to reduce HIV acquisition among pregnant and postpartum HIV-uninfected women in environments with high HIV incidence [27]. However, we need a greater understanding of the various concerns around taking PrEP during pregnancy and breastfeeding including the impact on the foetus, effect on the infant, effect on the pregnancy, and efficacy of the drug, and stigma around taking a pill associated with HIV, when HIV-uninfected. Importantly, the effectiveness of PrEP relies on adherence [12, 13]. Our study demonstrated that while PrEP knowledge was very low, however, hypothetical acceptability of PrEP was high. Most women interviewed were interested in learning more about PrEP to prevent HIV acquisition and control its effectiveness, especially because of the drug’s discretion. However, we identified barriers to PrEP uptake and adherence including substance use. We recommend research on how to deliver highly adaptable PrEP programs that can help reduce HIV incidence in this vulnerable population.

The World Health Organization (WHO) recently released oral PrEP guidelines for pregnant and breastfeeding women as “an additional prevention choice for people at substantial risk of HIV infection, as part of combination HIV prevention approaches”, defining “substantial risk” as HIV incidence >3 per 100 person-years in the absence of PrEP [28]. However, prior PrEP efficacy studies ensured that women used contraception during the study period and stopped PrEP provision when women became pregnant. Previous studies did not find adverse effects among infants who were exposed to TDF or emtricitabine (FTC) when the medications were taken as part of a treatment regimen for HIV-infected women during pregnancy or during breastfeeding (for which data suggest limited drug exposure [29–31]. PrEP adherence has been low during clinical trials of women. In the PrEP Trial for HIV Prevention among African Women (FEM-PrEP) and Vaginal and Oral Interventions to Control the Epidemic (VOICE) trials, PrEP was not effective at preventing HIV acquisition among women in Eastern and Southern Africa because of low drug exposure, presumably due to poor adherence [12, 13] One recent study in Kenya found that 34% of HIV-uninfected pregnant women accepted PrEP counselling in ANC and of those, 73% initiated PrEP on the same day. Factors associated with PrEP uptake included women with: a known HIV-infected partner, >1 partner, fewer fears about starting PrEP, and in polygamous marriages [32].

The limitations of this study include that we only asked about hypothetical, instead of real, use of PrEP. Surely there will be differences in PrEP acceptability once presented with the opportunity to take the drug for HIV prevention. The transferability of the findings to other groups may also be a limitation as our sample was limited to one facility among pregnant women who were relatively healthy compared to hospital births, but healthier and potentially more educated than women who may give birth at home.

## Conclusion

Our study assessed risk behaviours during pregnancy and post-partum periods and explored knowledge and hypothetical acceptability of PrEP among HIV-uninfected women. Women reported substantial barriers to HIV prevention including lack of control over sex and condom use, low perceived risk (especially after alcohol consumption), and socio-cultural beliefs including that frequent condomless sex is good for the baby during pregnancy, but may be risky for the baby after birth. Risk perception and actual risk for HIV acquisition was dynamic. While mothers recognized the need to protect themselves from HIV acquisition during pregnant, they reported increases in risky sexual behaviors, including frequent condomless sex throughout pregnancy. Mothers had low awareness of PrEP but expressed willingness to use PrEP because of the lack of control over their sexual risk and desire to prevent perinatal HIV transmission. These data have critical implications for design of programs that seek to deliver PrEP to pregnant women including the need for counselling to address the potential risk for the infant when taking PrEP, and how best to take PrEP when sexual activity is constantly changing during pregnancy and postpartum periods. More research is needed to understand how best to deliver PrEP counselling and services to pregnant and breastfeeding women who face changing sexual risk behaviours.
